# Linker-Extended Native Cyanovirin-N Facilitates PEGylation and Potently Inhibits HIV-1 by Targeting the Glycan Ligand

**DOI:** 10.1371/journal.pone.0086455

**Published:** 2014-01-27

**Authors:** Jia Chen, Dane Huang, Wei Chen, Chaowan Guo, Bo Wei, Chongchao Wu, Zhou Peng, Jun Fan, Zhibo Hou, Yongsheng Fang, Yifei Wang, Kaio Kitazato, Guoying Yu, Chunbin Zou, Chuiwen Qian, Sheng Xiong

**Affiliations:** 1 Institute of Biomedicine & National Engineering Research Center of Genetic Medicine, Jinan University, Guangzhou, Guangdong, China; 2 School of Pharmaceutical Sciences & Institute of Human Virology, Sun Yat-sen University, Guangzhou, Guangdong, China; 3 Department of Molecular Microbiology and Immunology, Nagasaki University, Nagasaki City, Nagasaki Prefecture, Japan; 4 Laboratory of Virus Control, Institute for Virus Research, Kyoto University, Kyoto, Japan; 5 Department of Medicine, University of Pittsburgh, Pittsburgh, Pennsylvania, United States of America; 6 Guangdong Provincial Institutes of Traditional Chinese Medicine, Guangzhou, Guangdong, China; Alexion Pharmaceuticals, United States of America

## Abstract

Cyanovirin-N (CVN) potently inhibits human immunodeficiency virus type 1 (HIV-1) infection, but both cytotoxicity and immunogenicity have hindered the translation of this protein into a viable therapeutic. A molecular docking analysis suggested that up to 12 residues were involved in the interaction of the reverse parallel CVN dimer with the oligosaccharide targets, among which Leu-1 was the most prominent hot spot residue. This finding provided a possible explanation for the lack of anti-HIV-1 activity observed with N-terminal PEGylated CVN. Therefore, linker-CVN (LCVN) was designed as a CVN derivative with a flexible and hydrophilic linker (Gly_4_Ser)_3_ at the N-terminus. The N-terminal α-amine of LCVN was PEGylated to create 10 K PEG-aldehyde (ALD)-LCVN. LCVN and 10 K PEG-ALD-LCVN retained the specificity and affinity of CVN for high mannose N-glycans. Moreover, LCVN exhibited significant anti-HIV-1 activity with attenuated cytotoxicity in the HaCaT keratinocyte cell line and MT-4 T lymphocyte cell lines. 10 K PEG-ALD-LCVN also efficiently inactivated HIV-1 with remarkably decreased cytotoxicity and pronounced cell-to-cell fusion inhibitory activity *in vitro*. The linker-extended CVN and the mono-PEGylated derivative were determined to be promising candidates for the development of an anti-HIV-1 agent. This derivatization approach provided a model for the PEGylation of biologic candidates without introducing point mutations.

## Introduction

Currently, over 30 million people worldwide are infected with human immunodeficiency virus type 1 (HIV-1) and 1.5 million-1.9 million people died from AIDS-related causes at the end of 2011; approximately 2.2 million-2.8 million people become infected each year, of whom 95% live in low- and middle-income countries [1], [2]. Microbicides are promising alternative agents for the prevention of HIV-1 transmission [3]. Cyanovirin-N (CVN), a protein originally isolated from the freshwater cyanobacterium *Nostoc ellipsosporum,* exhibits specific and potent anti-HIV activity by binding with high affinity to the glycans present on gp120 and gp41 [4], [5]. CVN irreversibly inactivates both laboratory-adapted and wild type HIV-1 strains during the viral entry stage. The antiviral effects of CVN also include the inhibition of cell-to-cell fusion, virus-to-cell fusion and cell-to-cell transmission [6]. CVN has generated interest as a promising new generation of microbicides characterized by specific and potent activity, a novel mechanism of action and unusual physicochemical stability.

CVN may be useful in two different clinical applications, either as a targeting agent or as a topical microbicide, to prevent the sexual transmission of HIV-1 by providing a method for female control over the HIV/AIDS epidemic [7]. Because of its cyanobacterial origins, CVN exhibits the limitations that are typical of such proteins in pharmaceutical applications, including a short plasma half-life, proteolysis and immunogenicity. Polyethylene glycol (PEG) is a well-studied polymer that is utilized as a covalent modification on biological macromolecules to improve biological compatibility by attenuating both immunogenicity and toxicity, to increase the half-life and to alter the biodistribution [8].

Although the literature has mainly focused on site-selective PEGylation that generates a single isomer, thereby increasing the homogeneity and facilitating the preservation of bioactivity, site-specific PEGylation at the N-terminus or on random amines on the side chains of CVN has resulted in inactive molecules [7], [9]. The only PEGylated version of CVN that is bioactive is the mutant Q62C, in which glutamine 62 was replaced with a cysteine, and the extra free sulfhydryl was site-specifically PEGylated with maleimide-activated PEG [7]. The *in vitro* anti-HIV-1 activity of the Q62C mutant was approximately 50% that of wild type (WT) CVN. The 20 kDa PEG-CVN Q62C conjugate demonstrated approximately 80% of the activity observed with CVN WT. The 30 kDa conjugate had nearly no activity. From these reported data, we hypothesized that N-terminal residues and certain lysine residues might exist in or near the glycan binding sites of CVN.

To confirm this hypothesis, molecular docking and experimental approaches were utilized to investigate the binding selectivity of CVN to oligosaccharides with various structures. The protein-ligand complexes of CVN 3GXY with high mannose N-glycans were also docked and analyzed to further characterize the hot spot residues in CVN. This structure-function relationship study suggested that Leu-1 in the N terminus was the most important hot spot residue for binding to Man_7−9_GlcNAc_2_ glycans. Therefore, a rational PEGylation process was designed to avoid blocking the N-terminal hot spot residues.

The well documented (Gly_4_Ser)_3_ molecule is a flexible hydrophilic linker peptide that has been utilized to fuse 2 independent polypeptides into a protein with multiple domains and functions [10]. Based on the merits of the (Gly_4_Ser)_3_ linker peptide and the conundrum of CVN PEGylation, we extended the N-terminus of CVN with (Gly_4_Ser)_3_ to create linker-CVN (LCVN) and performed site-specific PEGylation of LCVN at the N-terminal amine group using mPEG-aldehyde (ALD). We hypothesized that this PEG-linker-CVN might preserve the bioactivity of CVN by separating the large PEG group from the CVN active site as well as facilitate the preparation of highly homogenous PEGylated products. This strategy avoided introducing a point mutation into the primary sequence of CVN that could alter its bioactivity.

There is no sequence homology greater than 8 contiguous amino acids or 20% of the total sequence between CVN and any other known proteins. The extremely low sequence homology in addition to the 2 intramolecular disulfide bonds in CVN makes the artificial production of this protein in *Escherichia coli* (*E. coli*) difficult [6]. In a previous study, biologically functional CVN was efficiently expressed in the cytoplasm of *E. coli* after fusion to small ubiquitin-related modifier (SUMO) coupled with a hexahistidine tag [11]. Utilizing this strategy, the fusion gene *his_6_-sumo-linker-cvn* was constructed to efficiently produce soluble LCVN in *E. coli*. The N-terminal PEGylation of LCVN was performed to create 10 K PEG-ALD-LCVN, a site-specific PEG conjugate of LCVN. Subsequently, the gp120, gp41 and oligosaccharide binding characteristics of LCVN and 10 K PEG-ALD-LCVN were evaluated. The anti-HIV-1 activity and cytotoxicity of these 2 CVN derivatives were determined by MTT and syncytium-formation assays to elucidate the effects of the linker peptide on oligosaccharide binding and the anti-HIV-1 activity of CVN and to explore the feasibility of site-specific PEGylation of pharmaceutical proteins via the (Gly_4_Ser)_3_ extension.

## Results

### CVN Targeting to 24 Potential Oligosaccharides that were Selected from a Pool of 6 Types of N-glycans by Molecular Docking

**Figure 1:**
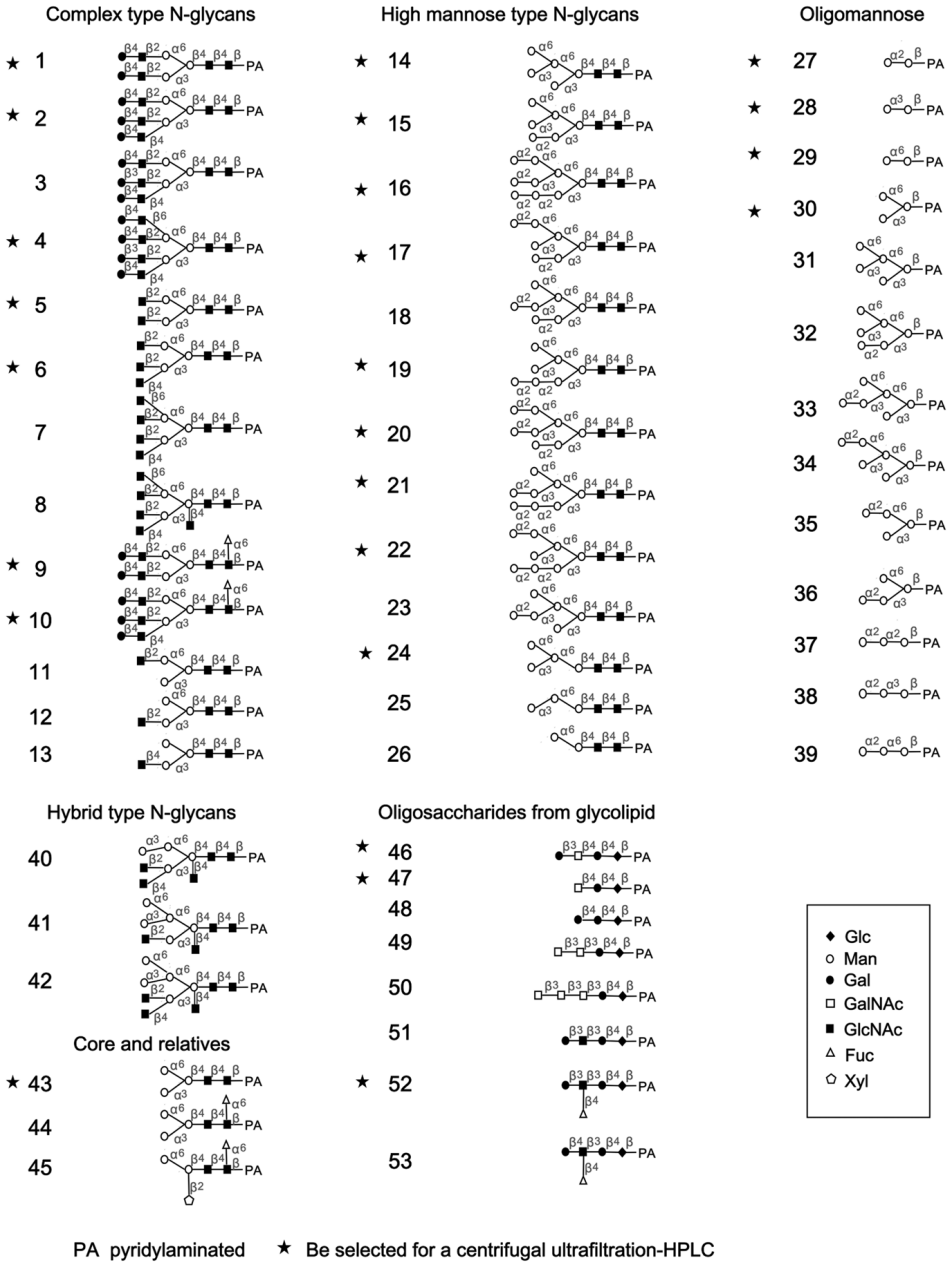
The PA-oligosaccharide structures utilized for the molecular docking and experimental target binding assays with LCVNs. A panel of 53 oligosaccharides was selected to represent the diverse carbohydrate structures. All these oligosaccharides were utilized in the docking experiments. After the docking simulation, 24 oligosaccharides (indicated by asterisks) were selected for further analysis by the centrifugal ultrafiltration-HPLC assay.

Molecular docking was performed to determine the binding selectivity of CVN for oligosaccharides with various structures and to clarify the binding modes. The crystallization data for CVN (Figure S1) (http://www.rcsb.org/pdb) was utilized to dock 53 oligosaccharide targets that were selected to represent 6 types of carbohydrate structures. These oligosaccharides included 13 complex N-glycans, 13 high mannose N-glycans, 13 branched and linear oligomannoses, 3 hybrid N-glycans, 3 N-glycans with a core pentasaccharide or related moiety and 8 oligosaccharides originating from glycolipids (Figure 1). The consensus scores (CS) for the 53 oligosaccharides are listed in Table 1. High scores indicated improved biological activity. Several complex and hybrid N-glycans exhibited a high CS. Oligosaccharides No. 1, 2, 4, 10 and 42 were characterized with a CS >0.5. Most high mannose N-glycans (No. 14–26) had a CS between 0.2 and 0.5. Several oligomannose moieties had a CS of zero.

**Table 1:** MOE docking values for 53 oligosaccharides in the training set.

Number	Consensus score	x	y	Number	Consensus score	x	y
Complex type				29	0.00	−45.26	−21.92
1	0.50	−74.17	−36.92	30	0.01	−46.64	−22.00
2	0.64	−85.65	−35.73	31	0.09	−55.72	−27.16
3	0.40	−73.56	−33.71	32	0.09	−58.58	−25.52
4	0.87	−84.39	−42.26	33	0.07	−56.84	−24.86
6	0.28	−64.58	−33.62	34	0.07	−58.52	−24.73
7	0.31	−77.59	−29.06	35	0.05	−52.76	−25.31
8	0.36	−84.63	−28.71	36	0.02	−48.68	−23.08
9	0.19	−68.80	−27.49	37	0.00	−44.68	−21.35
10	0.69	−90.33	−35.18	38	0.00	−47.01	−19.31
11	0.17	−67.25	−26.93	39	0.02	−48.96	−23.47
12	0.11	−59.78	−26.89				
13	0.28	−74.23	−29.28	Hybrid type			
High-mannose type				40	0.27	−68.94	−30.65
14	0.23	−63.13	−32.25	41	0.25	−69.04	−29.78
15	0.18	−67.32	−27.55	42	0.68	−79.95	−39.30
16	0.25	−67.67	−30.66	Core and relatives			
17	0.27	−64.41	−33.04	43	0.12	−63.93	−25.68
18	0.26	−69.41	−30.21	44	0.09	−61.16	−25.05
19	0.43	−77.20	−33.21	45	0.05	−57.87	−23.34
20	0.29	−75.00	−29.31	Oligosaccharides from glycolipids			
21	0.25	−71.72	−28.58	46	0.04	−54.39	−23.50
22	0.35	−71.32	−32.77	47	0.01	−46.37	−22.41
24	0.25	−69.83	−29.67	48	0.00	−53.08	−19.50
25	0.14	−60.42	−28.27	49	0.10	−62.76	−24.86
26	0.05	−55.69	−24.21	50	0.14	−60.98	−28.20
Oligomannoses				51	0.04	−59.08	−21.89
27	0.00	−43.73	−21.50	52	0.07	−57.67	−24.55
28	0.00	−44.16	−25.87	53	0.07	−56.60	−22.76

x, the docking energy for 3GXY;

y, the docking energy for 2PYS.

To characterize the CVN binding potential, dozens of high-scoring and commercially available oligosaccharides were selected from each oligosaccharide category for further investigation. According to this priority principle, 7 oligosaccharides (No. 1–2, 4–6, 9 and 10) that belonged to the maximum CS group were selected to represent the complex N-glycans. Nine oligosaccharides (No. 14–17, 19–22 and 24) that belonged to the medium CS group were selected to represent the high mannose N-glycans. From all the branched and linear oligomannoses in the minimum CS group, glycans No. 27–30 and 43 were selected to represent N-glycans with a pentasaccharide core, and No. 46–47 and 52 represented oligosaccharides originating from glycolipids. In total, 24 oligosaccharides (asterisks, Figure 1) were selected to represent the diverse carbohydrate structures in the centrifugal ultrafiltration-HPLC assays.

### Specific Recognition of CVN by Non-reduced Terminal Manα_1−2_Man Residues in Man_7−9_GlcNAc_2_ Glycans

**Figure 2:**
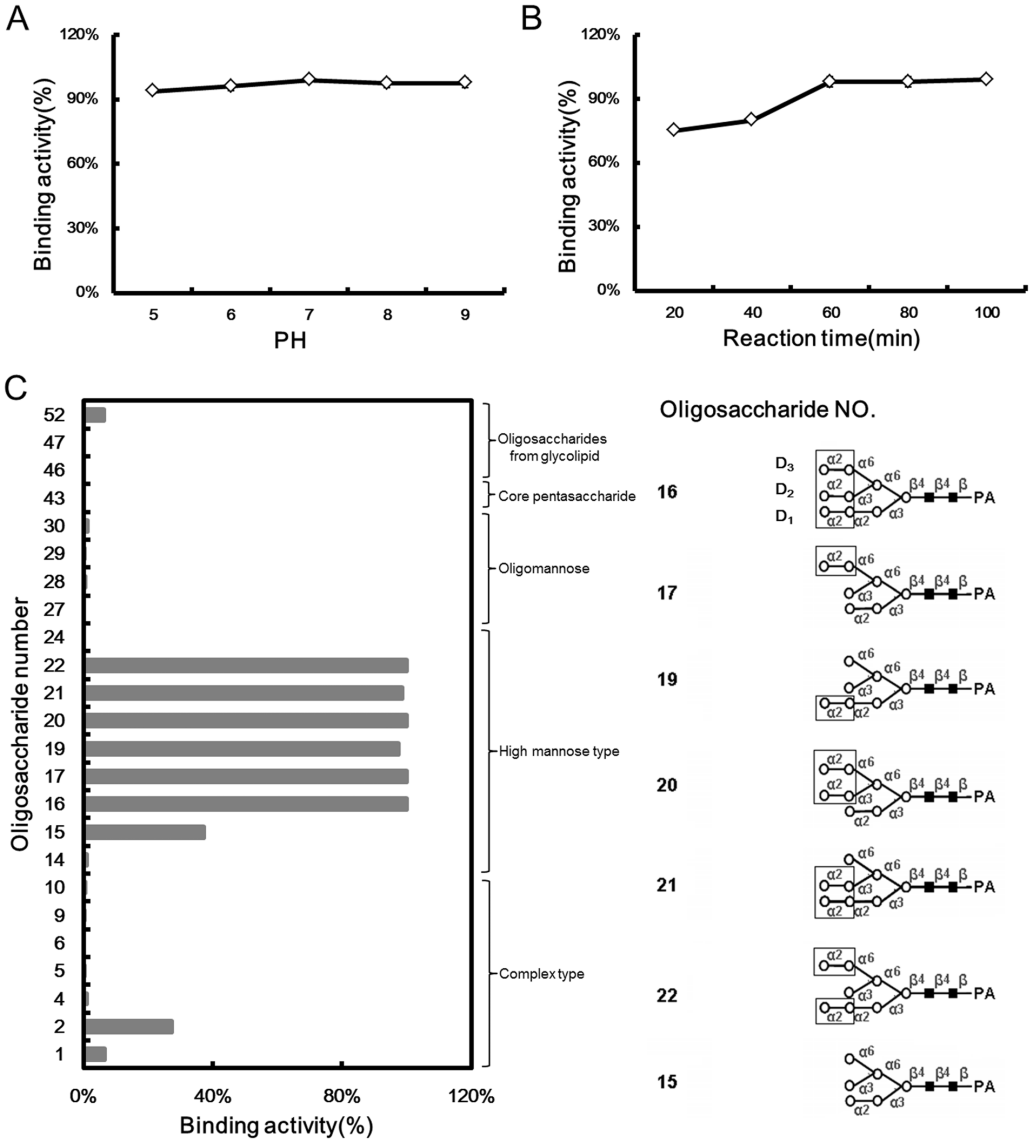
Binding specificities of CVN to PA-oligosaccharides. The optimum (**A**) pH and (**B**) reaction time for CVN binding to PA-heptasaccharide (No. 19, Figure 1) were determined via centrifugal ultrafiltration-HPLC, and subsequently the (**C**) binding activity (specificity) of CVN and the 24 oligosaccharides was measured. The right panel in (**C**) depicts the non-reducing terminal Manα_1−2_Man moieties in the D1, D2 or D3 arms of the Man_7−9_GlcNAc_2_ glycans that participated in the binding. Two independent experiments were performed for each PA-oligosaccharide, and the binding activity is presented as the average of the duplicate assays.

To verify the MOE docking data and evaluate the minimum oligosaccharide structure required for high-affinity binding to CVN, centrifugal ultrafiltration-HPLC was performed using fluorescence-labeled oligosaccharides (PA-oligosaccharides). The structures of the 24 oligosaccharides utilized in this study are indicated with asterisks in Figure 1. Before testing LCVN binding to the selected oligosaccharides, the optimum pH for the binding assay was determined using PA-heptasaccharide (No. 19, Figure 1) in 50 mM 2-(N-morpholino)ethanesulfonic acid (MES, pH 5.0), 50 mM sodium phosphate (pH 6.0) and 50 mM Tris-HCl (pH 7.0, 8.0 or 9.0). Similar assays were performed to optimize the reaction time from 20 to 100 min in 50 mM Tris-HCl (pH 7.0). Maximal CVN-oligosaccharide binding was achieved at pH 7.0–9.0 (Figure 2A) after a 60 min incubation period. The binding of CVN to its targets was stable after incubating for 60 min or longer (Figure 2B). Therefore, the binding of CVN to the selected PA-oligosaccharides was assayed at room temperature for 60 min at pH 7.0.

CVN exclusively bound to high mannose N-glycans (No. 15–22) without recognizing other oligosaccharides, including oligomannose (No. 27–30), complex N-glycans (No. 1–10) and oligosaccharides with a pentasaccharide core (No. 43) (Figure 2C). Glycolipid oligosaccharides (No. 46, 47 and 52) did not interact with CVN. The high mannose N-glycans (No. 16–17 and 19–22) bound to CVN with a binding ratio greater than 85% (Figure 2C). CVN bound with high affinity to Man_7_GlcNAc_2_ with 1 non-reduced terminal Manα_1–2_Man moiety in the D1 or D3 arm (No. 17 and 19). Man_8_GlcNAc_2_ with 2 exposed Manα_1–2_Man moieties in the D1, D2 or D3 arms (No. 20–22) or Man_9_GlcNAc_2_ with 3 Manα_1–2_Man moieties (No. 16) exhibited a strong interaction with CVN. The binding ratio decreased to 37% for oligosaccharide No. 15, which only has 1 non-reducing terminal Manα_1−2_Man moiety in the D1 arm provided by Man_6_GlcNAc_2_. These data clearly suggested that CVN exhibited strict specificity for high mannose N-glycans by recognizing the extended carbohydrate structure of the non-reducing terminal Manα_1−2_Man moieties in the D1, D2 or D3 arms provided by Man_7−9_GlcNAc_2_ (No. 16–17 and 19–22).

### Oligosaccharide Manα_1−2_Man Binding to the Hot Spot Residues was Critical for the Oligosaccharide-CVN Interaction

**Figure 3:**
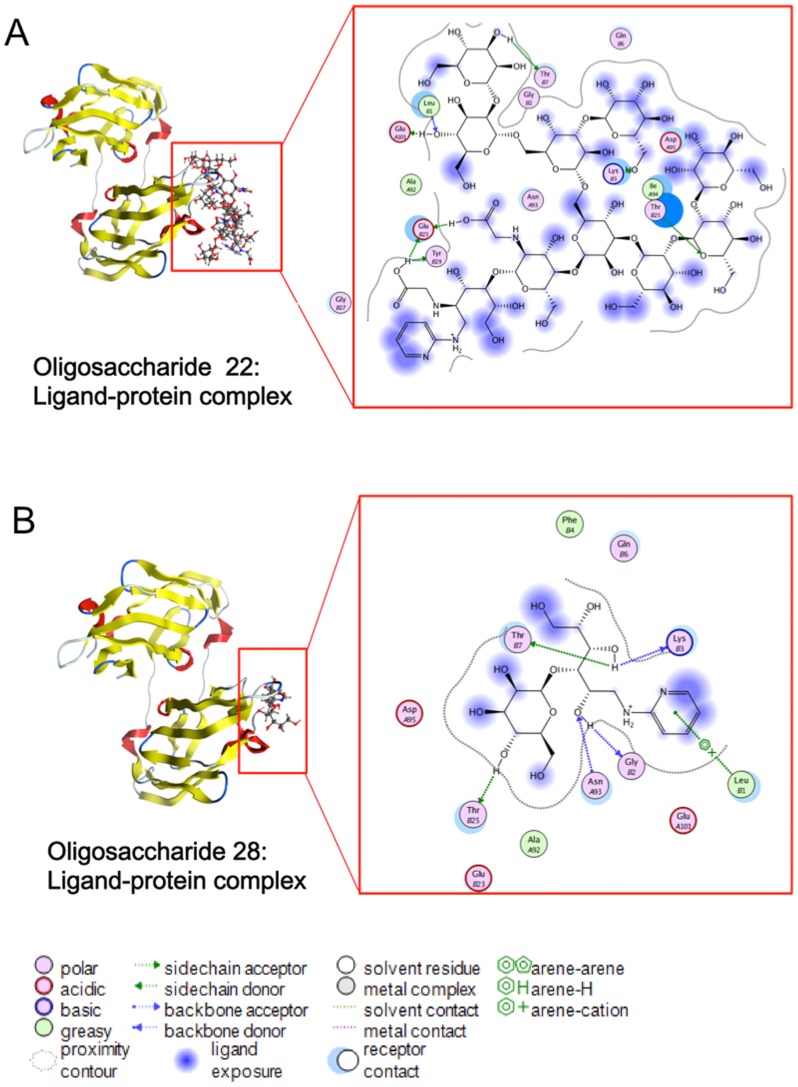
The mode of CVN binding to oligosaccharides. An overview of the mode of CVN binding to oligosaccharides (**A**) No. 22 and (**B**) No. 28 is presented. The fuzzy blue blob indicates ligand exposure to the solvent. For oligosaccharide 22, which exhibited strong binding, Leu-1, Lys-3, Thy-7, Glu-23, Thr-25, Tyr-29 and Glu-101 were involved in the protein-ligand interaction. For oligosaccharide No. 28, which was characterized by weak binding, the docking simulation indicated that hydrogen bonds formed between the ligand and Leu-1, Gly-2, Lys-3, Thr-7, Thr-25 and Asn-93.

To characterize the structural interactions between CVN and its ligands, oligosaccharides No. 22 and 28 were selected to represent an active and a less active group, respectively, for further analysis. The active pocket of CVN 3GXY is located in the gap between the β sheet of chains A and B. The extended structure of the non-reducing terminal mannose moieties in the oligosaccharide bound to the active pocket of CVN, whereas the other part of the oligosaccharide was exposed outside of the pocket (Figure 3). An overview of the protein-ligand interactions for oligosaccharides No. 22 and 28 is presented in Figure 3. For oligosaccharide No. 22, hydrogen bonds formed between the ligand and Leu-1, Lys-3, Thy-7, Glu-23, Thr-25, Tyr-29 and Glu-101 in the active site (Figure 3A); for oligosaccharide 28, hydrogen bonds were present between the ligand and Leu-1, Gly-2, Lys-3, Thr-7, Thr-25 and Asn-93 (Figure 3B). Although both the active and the less active oligosaccharides interacted with 6 residues in CVN, the binding energy for oligosaccharide No. 22 was −71.32 kcal/mol, which was lower than that for oligosaccharide No. 28 (−44.16 kcal/mol). The multiple hydrogen bond interactions and the lower binding energy for the oligosaccharide No. 22-CVN complex corresponded with the greater activity of this particular oligosaccharide.

Even with the differences in binding energy, oligosaccharides No. 22 and 28 interacted with the same 4 amino acids, Leu-1, Lys-3, Thy-7 and Thr-25 (Figure 3). For oligosaccharide No. 22, 5 amino acids (Leu-1, Lys-3, Thr-7, Thr-25 and Glu-101) interacted with the extended non-reducing terminal mannose moieties. In contrast, only 1 residue (Thr-25) interacted with the mannose of oligosaccharide No. 28, indicating that the multiple mannoses interacting with multiple residues contributed to the robust activity of oligosaccharide No. 22.

**Figure 4:**
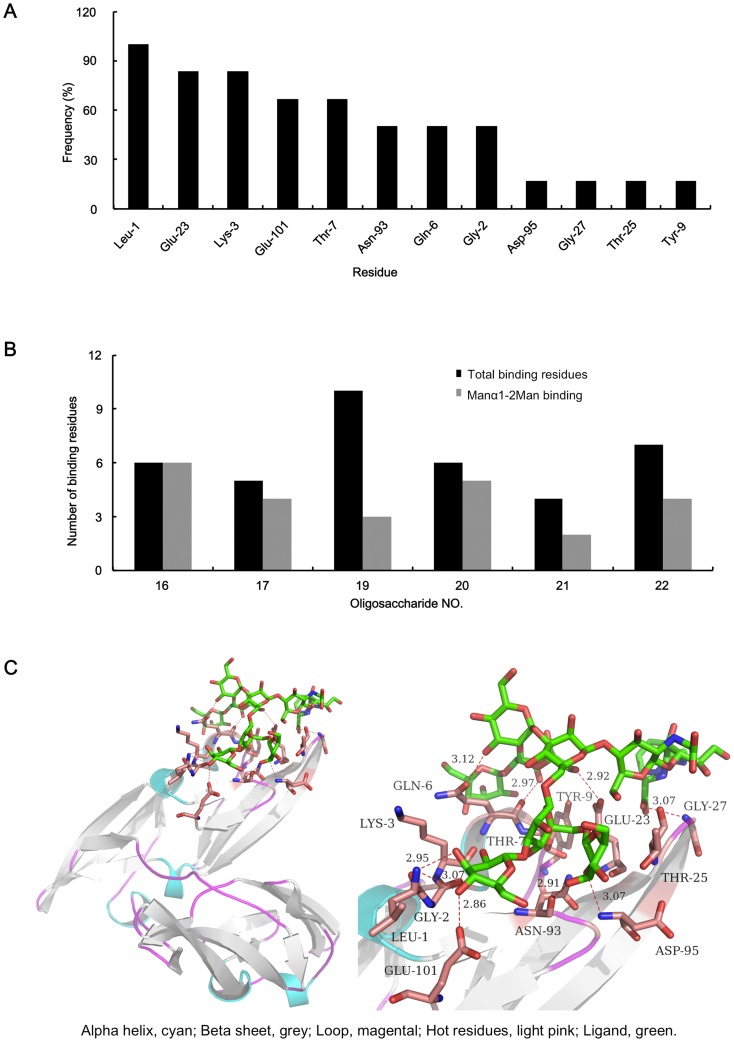
Characterization of CVN binding to high mannose oligosaccharides. (**A**) The frequencies of the hot spot residues that target the 6 high mannose oligosaccharides were calculated. Leu-1 represents the N-terminal leucine in the B chain, which was involved in oligosaccharide binding with a frequency of 100%. (**B**) The total number of targeting residues (black column) and Manα_1−2_Man binding residues (grey column) for CVN binding to the 6 oligosaccharides is presented. (**C**) The 3D model of CVN 3GXY binding to oligosaccharide No. 19 is illustrated. The 10 residues in CVN that were involved in the binding are colored in light pink, and their ligands are depicted in green. The hydrogen bonds are illustrated as dashed light pink lines.

To further characterize the hot spot residues in CVN and its derivatives, protein-ligand complexes of CVN 3GXY with all 6 high mannose N-glycans (No. 16–17 and 19–22) were docked and analyzed by MOE. The frequencies at which the hot spot residues were directly involved in the interactions are presented in Figure 4A. In total, 12 amino acids (Leu-1, Gly-2, Lys-3, Gln-6, Thr-7, Tyr-9, Glu-23, Thr-25, Gly-27, Asn-93, Asp-95 and Glu-101) in CVN were involved in binding to oligosaccharide ligands. Most of these residues could form hydrogen bonds with the ligands. All 6 oligosaccharides bound to Leu-1, and over half of the ligands bound to Gly-2, Lys-3, Gln-6, Glu-23, Asn-93 and Glu-101. The 3D structure formed by the 12 hot spot residues (the binding residues) was defined as a new binding pocket in CVN for oligosaccharides. This binding pocket could be utilized as a reference for further molecular docking studies to select novel ligands.

To illustrate the type of mannose structure that was specifically targeted in the oligosaccharide-CVN (3GXY) binding model and to evaluate the consistency with the centrifugal ultrafiltration-HPLC assay, all the binding moieties in the 6 oligosaccharides were analyzed and summarized as the number of total targeting residues in CVN and the number of Manα_1−2_Man-targeting residues involved in binding to each oligosaccharide (Figure 4B). For CVN, 4–10 residues bound to each oligosaccharide with 2–4 Manα_1−2_Man-targeting residues. In general, 63% of the binding occurred between hot spot residues and Manα_1−2_Man moieties. Oligosaccharide No. 19 was targeted by 10 amino acids, with 3 of these hot spot residues targeting the extended non-reducing terminal Manα_1−2_Man moieties. These data were consistent with the centrifugal ultrafiltration-HPLC study, suggesting that CVN specifically recognized the extended non-reducing terminal Manα_1−2_Man moieties provided by Man_7–9_GlcNAc_2_.

Because oligosaccharide No.19 was targeted by most hot spot residues, the 3D model of CVN 3GXY binding to this oligosaccharide is illustrated in Figure 4C. The 10 binding residues were Leu-1, Gly-2, Gln-6, Tyr-9, Glu-23, Thr-25, Gly-27, Asn-93, Asp-95 and Glu-101 (highlighted in light pink). The hydrogen bonds are indicated by dashed lines. Three of these hot spot residues, Gln-6, Tyr-9 and Glu-23, targeted the extended non-reducing terminal Manα_1−2_Man moieties. This model provided insight into the CVN-oligosaccharide interaction.

### Both LCVN and the PEGylated Product Retained the Specificity and Affinity of CVN for Man_7−9_GlcNAc_2_ Glycans

**Figure 5:**
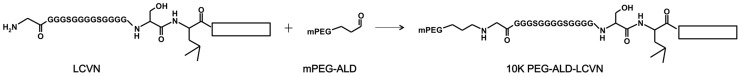
Structural schematics of LCVN and the PEGylated product. The N-terminal glycine of LCVN and the serine-leucine joint between the linker and the CVN sequence are indicated. The blank rectangle represents the residual polypeptide of CVN. 10 KD mPEG-ALD was selectively reacted with the N-terminal α-amine of LCVN at different pKa values to create 10 K PEG-ALD-LCVN.

The structure-function relationship study suggested that Leu-1 in the N terminus of CVN was the most important hot spot residue for binding to Man_7−9_GlcNAc_2_ glycans and that most of the N1 to N7 residues in the N terminus were involved in the binding. Therefore, LCVN, a CVN derivative with a (Gly_4_Ser)_3_ oligopeptide extension at the N terminus, was constructed to retain the integrity of the binding sites in CVN. The N-terminal α-amine of LCVN was PEGylated to create 10 K PEG-ALD-LCVN (Figure 5).

**Figure 6:**
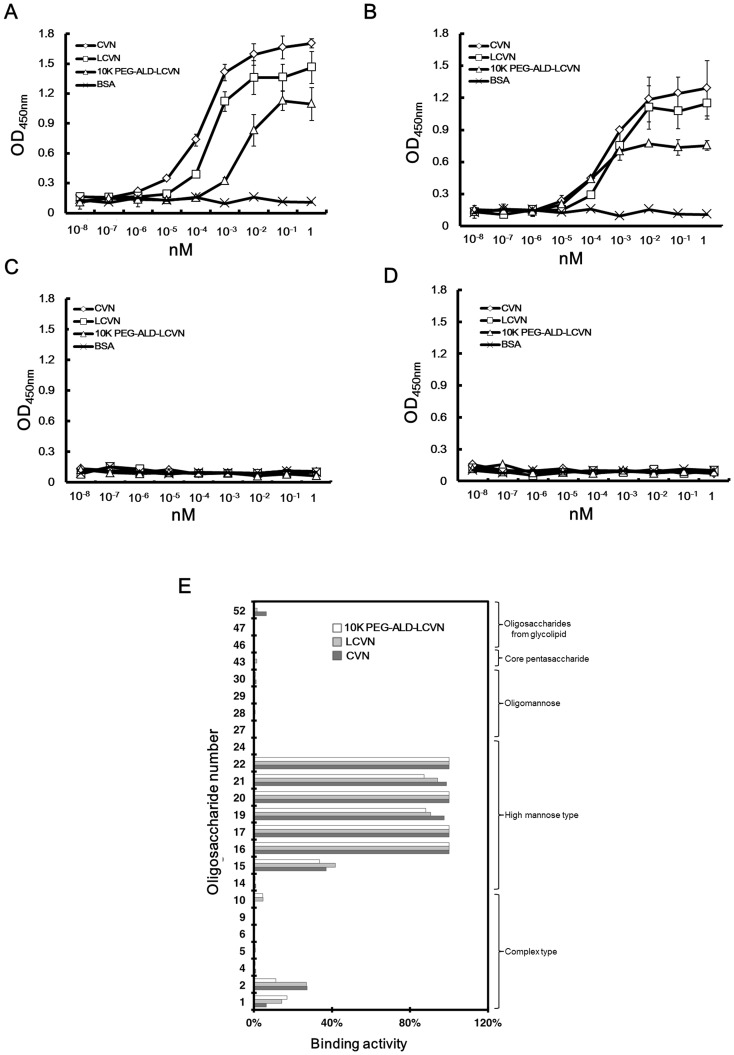
The binding of LCVNs to HIV-1 envelope proteins and oligosaccharides. The binding of LCVNs to glycosylated (**A**) gp41 and (**B**) gp120 and non-glycosylated (**C**) gp41 and (**D**) gp120 was determined by ELISA. CVN served as the positive control, and BSA was utilized as the negative control. The data points represent the mean±SD of independent triplicate experiments. (**E**) The LCVN binding activity (specificity) with the 24 oligosaccharides was assayed by centrifugal ultrafiltration-HPLC. Two independent experiments were performed for each PA-oligosaccharide, and the binding activity is presented as the average of the duplicate assays.

The gp120 and gp41 binding activities of LCVN and 10 K PEG-ALD-LCVN were determined to characterize their glycan binding ability. As a positive control, CVN bound to glycosylated gp41 (Figure 6A) and gp120 (Figure 6B) in a dose-dependent manner but did not exhibit any affinity for non-glycosylated gp41 (Figure 6C) or gp120 (Figure 6D). CVN bound more tightly to gp41 than to gp120. LCVN and 10 K PEG-ALD-LCVN had the same binding specificity to the glycosylated substrates (Figure 6A-6D), but their affinities were slightly decreased compared with CVN. These data suggested that both LCVN and 10 K PEG-ALD-LCVN maintained the glycan-specific binding of native CVN to both gp120 and gp41.

The binding of LCVN and 10 K PEG-ALD-LCVN to the 24 oligosaccharides (asterisks, Figure 1) selected to represent diverse carbohydrate structures was determined to characterize the glycan specificity. The centrifugal ultrafiltration-HPLC assays indicated that the 2 proteins exclusively recognized high mannose N-glycans (No. 15–22) over other types of oligosaccharides (No. 1–10, 27–30, 43, 46, 47 and 52) (Figure 6E). Although LCVN exhibited a slightly decreased affinity to high mannose N-glycans No. 19 and 21, it bound to high mannose N-glycans No. 16, 17, 20 and 22 with binding ratios of 100%. The binding ratio for oligosaccharide No. 15, which has 1 non-reducing terminal Manα_1−2_Man moiety in Man_6_GlcNAc_2_, and LCVN decreased to <40%, which corresponds with the data for CVN (Figures 6E and 2C). These data clearly suggested that both LCVN and 10 K PEG-ALD-LCVN retained the affinity of CVN for specific oligosaccharides. All 3 versions of CVN had identical target specificity and consistent binding potency to the extended carbohydrate structure of the non-reducing terminal Manα_1−2_Man moieties in the D1, D2 or D3 arms provided by Man_7−9_GlcNAc_2_ glycans.

### LCVN Cytotoxicity was Significantly Lower and was Further Attenuated by PEGylation

As promising microbicide candidates, CVN and its derivatives would be applied topically on human skin and/or mucosa. The HaCaT keratinocyte cell line and the MT-4 T lymphocyte cell line were utilized to evaluate the *in vitro* cytotoxicity of LCVN and its derivatives. Both LCVN and 10 K PEG-ALD-LCVN exhibited significantly less cytotoxicity than native CVN (Table 2). For HaCaT cells treated for 24 h, the CC_50_ values for LCVN and 10 K PEG-ALD-LCVN were 8.59±1.31 µM and >12.00 µM, respectively. The value for native CVN was 1.74±0.22 µM, suggesting that the cytotoxicity of LCVN was approximately 1/6 that of CVN. For 10 K PEG-ALD-LCVN, the cytotoxicity was <1/10 that of CVN. After treating the cells for 48 h, LCVN exhibited less cytotoxicity than CVN, and 10 K PEG-ALD-LCVN was approximately 1/6 as cytotoxic as native CVN. The MT-4 T lymphocyte cell line was sensitive to the various versions of CVN, with both LCVN and the PEGylated product exhibiting significantly reduced toxicity (Table 3). The cytotoxicity of LCVN was approximately 1/4 that for CVN. For 10 K PEG-ALD-LCVN, the ratio was 1/42. These data suggested that LCVN was remarkably less cytotoxic than native CVN and that the *in vitro* toxicity was further reduced by PEGylation. The addition of the 15-aa extension (linker) at the N-terminus of CVN decreased the cytotoxicity. After PEGylation, this toxicity decreased by approximately 40-fold. These results suggested that further examination of these modified proteins for potential anti-HIV activity would be beneficial.

**Table 2:** The cytotoxicity of LCVN and its PEGylated derivatives in HaCaT cells (mean±SD, n = 3).

	Cytotoxicity (CC_50_, µM)	
	24 h	48 h
LCVN	8.59±1.31	1.75±0.14
10 K PEG-ALD-LCVN	>12.00	6.11±0.29
CVN	1.74±0.22	1.18±0.09

CC_50_, 50% cell inhibitory concentration.

**Table 3:** The anti-HIV-1 activity and MT-4 cytotoxicity of LCVNs (mean±SD, n = 3).

	IC_50_ (nM)	CC_50_ (nM)	SI
LCVN	14.36±1.35	647.15±93.42	45.06
10 K PEG-ALD-LCVN	176.40±25.03	7181.07±861.72	40.71
CVN	21.83±2.79	169.56±13.63	7.77
AZT	36.55±5.64	>4482	>122.63

IC_50_, 50% viral inhibitory concentration;

CC_50_, 50% cell inhibitory concentration;

SI, the ratio of CC_50_ to IC_50_.

### LCVN Exhibited More Potent Anti-HIV-1 Activity in the Nanomolar Range

The anti-HIV activities of both LCVN and 10 K PEG-ALD-LCVN were determined by the WST-1 method. As presented in Table 3, LCVN and the PEGylated product protected MT-4 cells from infection with HIV-1/IIIB. The IC_50_ of LCVN was 14.36±1.35 nM and that for native CVN was 21.83±2.79 nM, suggesting that LCVN possessed more anti-HIV activity than native CVN. Although 10 K PEG-ALD-LCVN exhibited significantly less anti-HIV activity than both LCVN and CVN, the cytotoxicity to MT-4 cells was also significantly decreased. Considering activity and cytotoxicity, both LCVN and 10 K PEG-ALD-LCVN exhibited improved safety profiles. The SI values for LCVN and 10 K PEG-ALD-LCVN were approximately 5-fold higher than that for CVN. Among the 3 CVN derivatives, LCVN exhibited the most potent anti-HIV activity, the highest SI value and the lowest cytotoxicity. The derivative 10 K PEG-ALD-LCVN retained the potent anti-HIV activity of CVN in the nanomolar range and possessed the lowest cytotoxicity, which was similar to that of AZT.

### LCVN Exhibited Significantly Greater Fusion Inhibitory Activity than CVN

**Figure 7:**
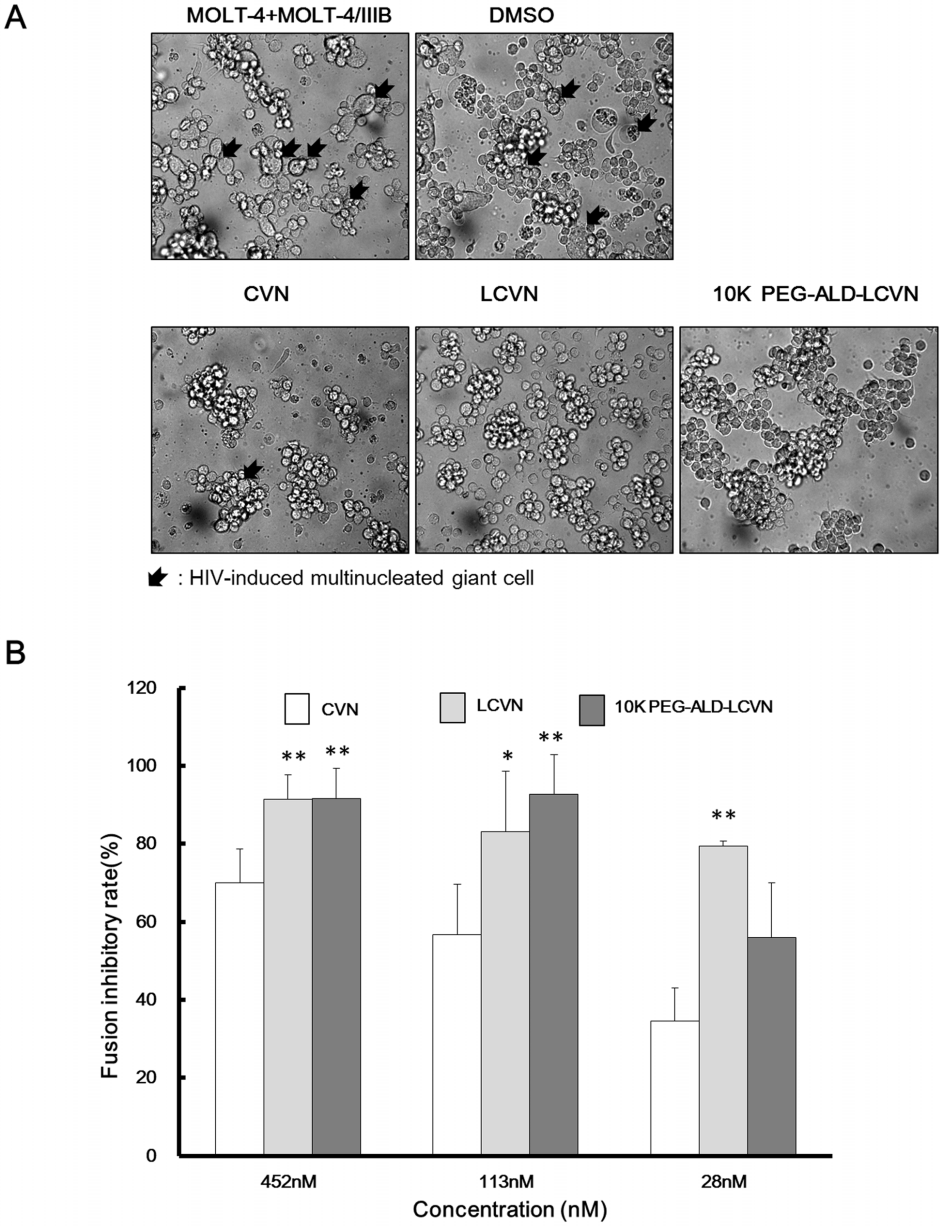
LCVN fusion inhibitory activity. (**A**) Phase-contrast micrographs were obtained 24 h after co-culturing normal MOLT-4 and HIV-1-positive cells in the presence of LCVN. The HIV-1-induced multinucleated giant cells are indicated by black arrows. (**B**) The relative fusion inhibition rates (%) for LCVN and its derivatives were calculated (**P<0.01 vs. CVN, * *P*<0.05 vs. CVN).

HIV-1 spreads efficiently, primarily via cell-to-cell fusion. To determine the fusion inhibitory activity of LCVN and its PEGylated conjugate, a cell-to-cell fusion assay was performed according to the method of Tochikura *et al*. [12]. In this syncytium formation assay, MOLT-4 cells were co-cultured with HIV-producing MOLT-4/IIIB cells for 24 h in the presence of LCVN or its derivatives. The results demonstrated that the fusion inhibitory activity of LCVN was significantly greater than that of CVN; PEGylation further enhanced this activity in the high and medium dose groups (Figure 7). In the lower dose group (28 nM), 10 K PEG-ALD-LCVN exhibited less inhibitory activity than LCVN, but its activity remained higher than that of CVN. These data confirmed the merits of enhancing bioactivity and attenuating toxicity by adding an N-terminal linker (LCVN) and suggested that the PEG groups in 10 K PEG-ALD-LCVN might interfere with the fusion of HIV-1-positive cells to normal ones by steric hindrance. This hypothesis and these data provided insight into the mechanism of HIV-1 transmission and will aid the discovery and development of novel fusion inhibitory compounds.

## Discussion

A microbicide must not damage the mucosa because such damage may increase the risk of HIV-1 infection. Significant efforts had been made to reduce the toxicity and increase the anti-HIV activity of microbicide candidates, such as CVN. In this study, the structure-function relationship for CVN was investigated to identify a more rational structure for a CVN derivative for further optimization. After the first docking of CVN 3GXY and 2PYS to the 53 oligosaccharides, most complex and hybrid N-glycans exhibited a high CS with low or no binding in the experimental assays. By analyzing the protein-ligand binding modes, the nitrogen in the oligosaccharide was determined to interact with the amino acids of CVN, accounting for the majority of the binding free energy. CVN did not interact with oligomannose (No. 27) and had a low affinity for Man_6_GlcNAc_2_ glycan (No. 15). These data suggested that the Man_5_GlcNAc_2_ core in N-glycans with 6–9 mannose moieties was essential for the interaction with CVN and its mutants. The terminal Manα_1−2_Man moieties and the conformation of the glycosidic linkage between the terminal disaccharide and the core residue(s) structure might be responsible for the observed selectivity.

Multiple experimental and computational methods, including crystallization, molecular dynamics (MD) simulations and point mutation studies, have been utilized to investigate the antiviral mechanisms of action and to optimize the structural features of CVN [13], [14], [15], [16], [17], [18]. In our experimental assays, CVN, LCVN and the PEGylated conjugate exclusively bound to high mannose N-glycans (No. 16–17 and 19–22) without recognizing other N-glycans. This was consistent with the docking analyses that suggested that 63% of the binding residues were formed by the Manα_1−2_Man moiety. Our data and a previous STD-NMR study suggested that both the terminal disaccharide and the reducing mannose residue influenced the affinity and the selectivity of interactions with CVN [19]. Furthermore, we discovered that CVN recognized Man_8_GlcNAc_2_ and Man_9_GlcNAc_2_ glycans with 2 or 3 reducing Manα_1−2_Man ends [17] and Man_7_GlcNAc_2_ glycans with 1 reducing end. In contrast with STD-NMR studies that utilized the di- and tri-mannoside substructures of Man-9 to determine the oligosaccharide specificity of CVN, we selected 5 different types of Man_7–9_GlcNAc_2_ glycans that represented the carbohydrate structures of gp120 [20]. These data could be utilized to determine the type of carbohydrate structures in gp120 that could be targeted by CVN and LCVNs with high affinity.

Thirteen residues, Gly-2, Lys-3, Thr-7, Glu-23, Asn-42, Asp-44, Ser-52, Asn-53, Thr-57, Lys-74, Gln-78, Asn-93 and Asp-95 (Table S1), interacted with the oligomannose ligands in the crystal data for CVN 3GXY, 3GXZ, 2PYS, 1IIY and 2RDK. As illustrated in Figure 4, molecular docking suggested that 12 residues were involved in the binding of CVN 3GXY/Z to the 6 high mannose N-glycans. Among these hot spot residues, Gly-2, Lys-3, Thr-7, Glu-23, Asn-93 and Asp-95 were identified in the crystal structures and the simulated oligosaccharide-CVN 3GXY/Z complexes.

The binding of the 6 N-glycans to the 3 parallel CVN dimers 2PYS, 1IIY and 2RDK was analyzed. The docking analysis suggested that 8 residues were hot spots: Glu-41, Asn-42, Ser-52, Asn-53, Glu-56, Thr-57, Lys-74 and Arg-76. Five residues, Asn-42, Ser-52, Asn-53, Thr-57 and Lys-74, were present in both the crystallography and the computational data. Among the residues from the docking data, Glu-41 was the most important hot spot residue, with a frequency of 83%, suggesting that Glu-41 might be one of the critical binding residues in the parallel CVN dimer; however, this residue was not present in the crystallographic protein-oligomannose complex. Among all the hot spot residues involved in binding, 55% of them bound to the Manα_1−2_Man moiety of the oligosaccharide (data not shown).

A comparison of the docking and the crystallography data for the 2 types of CVN dimers with the experimental glycan/oligosaccharide binding assay data suggested that (i) the simulations of the CVN-oligosaccharide complexes were highly consistent with the interaction mode suggested by crystallography and (ii) the simulation had high fidelity with the experimental assay. An analysis of the docking of CVN to the oligosaccharides suggested that (i) Leu-1, Gly-2, Lys-3, Thr-7, Glu-23, Asn-93 and Asp-95 were particularly important for the binding of reverse parallel CVN to its targets, with Leu-1 being the most predominant hot spot residue; and (ii) in parallel CVN dimers, Glu-41, Asn-42, Asn-53, Thr-57 and Lys-74 were the most important hot spot residues. This binding model provided a possible explanation for the reduction in bioactivity for N-terminal PEGylated CVN and also supported our strategy of utilizing PEGylation in conjunction with a linker to separate the large PEG group from the oligosaccharide binding site in CVN. Furthermore, it was deduced that CVN might be more inclined to form a reverse parallel structure in solution because N-terminal PEGylated CVN was reported to be fully inactive, but all the hot spot residues are located in the center of parallel CVN [7].

Gp120 is responsible for target cell tropism and viral attachment via an interaction with the cell surface receptor CD4 and the co-receptors CCR5 or CXCR4 [21], [22]. The binding of gp120 to its receptor and co-receptor induces a cascade of refolding events in gp41 that bring the viral and cell membranes together [23]. CVN binds with high affinity to glycosylated gp120 and gp41. The stronger binding to gp41 than gp120 suggested that CVN interferes with the process of HIV receptor recognition and membrane fusion. In addition, CVN may act at the stage of membrane fusion by binding to gp41, thereby inhibiting the gp41 refolding events.

Based on the knowledge above, LCVN was designed and further modified at the N-terminus using a site-specific method and 10 KD mPEG-ALD to maintain the integrity of the binding sites in CVN. LCVN exhibited greater anti-HIV-1/IIIB activity in the MTT and fusion inhibitory assays and lower cytotoxicity than native CVN. The enhanced bioactivity of LCVNs may have resulted from (i) the (Gly_4_Ser)_3_ linker contributing to correct folding and the proper biological function of the linker-tagged protein [24]; (ii) the increased molecular weight and the enhanced thermostability that amplified the steric hindrance of LCVN, increasing the fusion inhibitory activity [25]; and (iii) the hydrophilicity of the flexible linker, which could interfere with the structural integrity of the viral envelope. It would be interesting to fully elucidate the mechanism by which LCVN exhibited enhanced anti-HIV-1 activity.

The anti-HIV-1/IIIB activity of 10 KD mPEG-ALD was significantly decreased in the WST-1 assay, but this LCVN derivative exhibited more potent fusion inhibitory activity than native CVN. WST-1 is a substrate that measures the metabolic activity of viable cells, so the WST-1 assay indirectly evaluates the anti-viral activity of a tested compound. The fusion inhibitory assay directly measures the antiviral activity of CVN because this assay simulates the actual process of HIV-1 transmission between normal and HIV-1-infected cells. Therefore, the fusion inhibitory assay is more pertinent for studying the antiviral mechanism of action of CVN. The fusion inhibitory activity of 10 K PEG-ALD-LCVN was greater than that of LCVN. These data strengthened the therapeutic potential for 10 K PEG-ALD-LCVN and suggested that the molecular weight of CVN and its derivatives might be crucial for antiviral activity. The steric hindrance effect might be involved in the antiviral mechanism of action of 10 K PEG-ALD-LCVN. Other groups have reported that derivatives with a higher molecular weight, such as the engineered CVN dimer, have greater antiviral activity than monomeric forms of CVN [26]. An interesting recent report demonstrated that fusions of CVN and the gp41 membrane-proximal external region (MPER) peptide joined by a (Gly_4_Ser)_x_ linker (where x is 4 or 8) induce specific, irreversible lysis of pseudotyped HIV-1 virions and fully infectious HIV-1 virions [27]. Both fusion components, CVN and MPER, are required for the cell-free virolysis of HIV-1. Considering the merits of the PEG-*linker*-CVN that we demonstrated here, it would be interesting to create a chimeric CVN derivative with N-terminal PEGylation and a C-terminal MPER fusion and to explore the potential of this novel agent, PEG-*linker*-CVN-*linker*-MPER, as a tri-acting virucidal entry inhibitor of HIV-1.

It would be prudent to test the anti-HIV-1 activity of LCVN and its PEGylated conjugate on additional HIV-1 strains. Here, we only utilized HIV-1/IIIB as a model strain to evaluate the potential of LCVN and the PEG-LCVN conjugate. It has been well validated that CVN irreversibly inactivates a broad range of laboratory-adapted HIV strains and clinical isolates with different tropisms at the nanomolar level [28], [30]. For example, the EC_50_ values for CVN against the HIV-1 R5 strains HIV-1(Ba-L), HIV-1(Ada-M) and HIV-1(89.6) are 17 nM, 1.7 nM and 36.8 nM, respectively [28], [29]. Our data demonstrated that LCVN and 10 K PEG-ALD-LCVN retained the specificity and potency of the anti-HIV-1 activity of CVN and suggested the therapeutic potential of these CVN derivatives against R5 and other HIV-1 strains.

## Conclusions

A linker-extended CVN derivative, LCVN, and its PEGylated product, 10 K PEG-ALD-LCVN, were rationally designed and constructed after molecular docking and experimental approaches. Twelve residues were determined to be involved in the targeting of the reverse parallel CVN dimer to oligosaccharide ligands, among which Leu-1 (the N-terminal leucine in the B chain of the CVN dimer) was the most important hot spot residue. Eight residues were suggested to interact with the oligosaccharides in the parallel CVN dimer, with Glu-41 being one of the most important hot spot residues. Both LCVN and 10 K PEG-ALD-LCVN retained the oligosaccharide specificity of CVN binding to high mannose N-glycans with >1 terminal Manα_1−2_Man moieties in gp120 and gp41. It was exciting that the CVN derivatives exhibited potent anti-HIV activity with remarkably decreased cytotoxicity. The improved biological compatibility of these 2 CVN derivatives suggested that these modifications could produce promising microbicide candidates and provide a template for a universal strategy for the PEGylation of biologic candidates without introducing point mutations. The CVN-oligosaccharide interaction analysis provided a possible explanation for the loss of anti-HIV-1 activity with N-terminal PEGylated CVN and suggested the dominant conformation of the CVN dimer in solution.

## Materials and Methods

### Chemicals, Reagents and Media

All the chemicals and reagents were obtained from Sigma (St. Louis, MO, USA) unless otherwise stated. All the media and supplements, including fetal bovine serum (FBS), were purchased from Invitrogen (New York, NY, USA) unless otherwise stated. Recombinant LCVN and the PEGylated product 10 K PEG-ALD-LCVN (Figure 5) were prepared in-house by a process modified from Gao *et al* [11].

### Cell Culture

The immortal human HaCaT keratinocyte cell line, purchased from the China Center for Type Culture Collection (Wuhan University, Wuhan, China), was propagated in Eagle’s minimal essential medium (MEM) supplemented with 10% FBS, 1.0 mM sodium pyruvate, 0.1 mM non-essential amino acids and 1.5 g/L sodium bicarbonate. The MT-4 T lymphocyte cell line (NIH AIDS Reagent Program, Germantown, MD, USA) was cultured in RPMI 1640 medium containing 10% FBS and 0.22% sodium bicarbonate. The cells were cultured at 37°C in a humidified atmosphere with 5% CO_2_.

### Enzyme-linked Immunosorbent Assay (ELISA)

The target binding activity of LCVN to HIV-1 gp120 and gp41 was determined using an ELISA-like assay as described previously [30]. Briefly, 100 ng of gp120 or gp41 derived from HIV-1^HXBc2^ (BPB, Beijing, China) was coated onto a 96-well plate, which was subsequently incubated with serially diluted LCVN or 10 K PEG-ALD-LCVN. The bound proteins were detected using a rabbit-anti-CVN polyclonal antibody (1∶10000, in-house preparation) and goat-anti-rabbit IgG-HRP (1∶3000) followed by chromogenic development with 3, 3′, 5, 5′-tetramethylbenzidine (TMB) at λ_450_.

### Molecular Docking

CVN exists predominantly as a monomer in solution and as a domain-swapped dimer in crystals, producing both parallel (head-to-head fashion) and reverse-parallel dimer conformations. The crystal structures of parallel CVN (2PYS) and reverse-parallel CVN (3GXY) (Figure S1) were downloaded from the RCSB Protein Data Bank (http://www.rcsb.org/pdb) [31]. To determine the optimum scoring function, docking experiments were performed for all the protein-ligand complexes using 3 molecular docking platforms (Flex_X [32], CDOCKER (DS 2.1, Accelrys) [33] and MOE [32]). The active sites were identified using the crystallographic ligand for all the datasets. All the docking experiments reported here were performed with the default parameters. Based on the ligand-protein binding energy, the 30 top-ranked docking poses were retained for further study.

The optimum docking program for CVN was selected using the root-mean-square deviation (RMSD) and the scores of the re-docking of the ligands to the known CVN crystal structures. After re-docking the ligands to 2PYS and 3GXY, the RMSD values with Flex_X ranged from 0.1 to 9.2, and the re-docking scores with CDOCKER were >0 kcal/mol. For MOE, the RMSD values were <1, and the docking scores were <−35.6 kcal/mol, indicating that MOE was the most appropriate program for CVN docking.

MOE has 2 docking placement methods, Alpha Triangle matcher and Proxy Triangle [34]. The active site was minimized using the AMBER 99 force field in MOE with the default parameters. All the oligosaccharides were docked, employing Triangle Matcher as the placement method and London dG as the first scoring function. The refinement was set to force field (AMBER 99), and the docked poses were energy-minimized in the receptor pocket. Affinity scoring was utilized to assess and rank the receptor-ligand complexes. A low docking score correlated with increased binding affinity.

To screen novel and bioactive targets of CVN, the 2D structures of 53 oligosaccharides were converted into 3D structures. With the energies minimized, the moieties were docked into the binding sites of 2PYS and 3GXY by MOE. A consensus score (C*S*_i_) was calculated from the normalized docking score of 3GXY (*X*_i_) and 2PYS (*Y*_i_) to objectively rank the 53 oligosaccharides with a high degree of confidence.
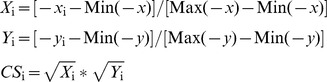



Function *X*_i_ is the normalized docking score of an oligosaccharide to 3GXY, and *x*_i_ is the (un-normalized) docking score of an oligosaccharide to 3GXY. Function *Y*_i_ is the normalized docking score of an oligosaccharide to 2PYS, and *y*_i_ is the (un-normalized) docking score of an oligosaccharide to 2PYS. Min(−*x*) and Min(−*y*) are the minimum scores among the values determined for the 53 oligosaccharides. Max(−*x*) and Max(−*y*) are the maximum scores among the values determined for the 53 oligosaccharides.

### Centrifugal Ultrafiltration-HPLC

A centrifugal ultrafiltration-HPLC assay was utilized to determine the oligosaccharide binding properties of the LCVNs as described by Katoh et al. [35]. Briefly, the LCVNs were incubated with pyridylaminated (PA)-oligosaccharides (Takara Bio Inc., Otsu, Shiga, Japan) in 50 mM Tris-HCl (pH 7.0) at room temperature for 60 min. The reaction mixture was centrifuged (14,000 xg for 15 min) in a centrifugal ultrafiltration tube (Sartorius Stedim, Boston, MA, USA) with a molecular weight cut-off value of 5,000 Daltons. The unbound PA-oligosaccharides (*O*_unbound_) and the total amount of added PA-oligosaccharides (*O*_added_) were quantified from the peak area detected on a TSKgel ODS 80™ column (4.6×150 mm) (Tosoh Corporation, Tokyo, Japan) at λ_320/400_ for the coupled fluorescence group. The bound PA oligosaccharide (*O*_bound_) was defined as the volume of *O*_added_ minus that of *O*_unbound_. The binding activity was expressed as the ratio of *O*_bound_ to *O*_added_ and presented as % binding.

### MTT Assay

The *in vitro* cytotoxicity of the LCVNs was determined via a 3-(4,5-dimethylthiazol-2-yl)-2,5-diphenyl tetrazolium bromide (MTT) assay in HaCaT cells. LCVNs were serially diluted from 12 µM to 0.375 µM, added to a monolayer of HaCaT cells in 96-well plates and incubated for either 24 or 48 h. The MTT solution was added for color development. The absorbance was measured at λ_570_/_630_, and the data were plotted to obtain the 50% cell inhibitory concentration (CC_50_).

### WST-1 Assay

The *in vitro* anti-HIV-1 activity of the LCVNs was determined using the water-soluble tetrazolium salt 2-(4-iodophenyl)-3-(4-nitrophenyl)-5-(2,4- disulfophenyl)-2H-tetrazolium (WST-1) in a P3 laboratory. For this assay, 2-fold dilutions of LCVNs were mixed with HIV-1/IIIB (100 TCID_50_/50 µL), added to MT-4 cells (10^4^ cells/100 µL/well) in 96-well microplates and incubated for 96 h. WST-1 was added to quantitate the number of viable cells at λ_450/650_. The cytotoxicities of the LCVNs in MT-4 cells were simultaneously determined in virus-free wells. Azidothymidine (AZT) was utilized as a positive control. The 50% inhibitory concentration (IC_50_), 50% cytotoxicity concentration (CC_50_) and selectivity index (SI, the ratio of CC_50_ to IC_50_) were determined.

### Cell-to-cell Fusion Assay

Cell-to-cell fusion assays and cell-to-cell virus transmission assays, also known as syncytium formation assays, were performed with a co-culture system comprised of MOLT-4 (ATCC® CRL-1582™, Manassas, VA, USA) and MOLT-4/IIIB cells as previously described [36]. MOLT-4/IIIB cells are chronically HIV-1-infected MOLT-4 cells that allow HIV-1 replication and induce syncytium formation between uninfected MOLT-4 cells.

MOLT-4 cells (2.5×10^5^/250 µL) and MOLT-4/IIIB cells (2.5×10^5^/250 µL) were mixed and seeded in 24-well plates (Falcon). LCVN and PEGylated LCVN were diluted 4-fold with RPMI-1640 containing 10% fetal calf serum and antibiotics. Individually cultured MOLT-4 and MOLT-4/IIIB cells and co-cultures of MOLT-4 and MOLT-4/IIIB cells without LCVN or PEGylated LCVN were utilized as controls, and co-cultured cells treated with LCVN or PEGylated LCVN at three different concentrations (452, 113 and 28 nM) were examined as the test conditions. Cytopathic effects indicated by the presence of syncytia formation were observed and recorded using a microscope after 24 h at 37°C with 5% CO_2_. The number of viable cells was determined by trypan blue dye exclusion, and the fusion index (FI) was calculated as the following: FI = 1−[cell number in a test well (MOLT-4+MOLT-4/IIIB)]/[cell number in control well (MOLT-4 only)]. The fusion inhibition rate (FIR) (%) = [1−(FI_T_/FI_C_)]×100 was also calculated, where FI_T_ was the fusion index of the test sample and FI_C_ was that of the co-cultured control [12].

## Supporting Information

Figure S1 The three-dimensional (3D) structures of CVN utilized in this study. 2PYS, 1IIY and 2RDK are parallel domain-swapped dimers of CVN, and 3GXY and 3GXZ are reverse-parallel domain-swapped dimers of CVN. The structure coordinates of the protein-ligand complexes were retrieved from the Protein Data Bank (PDB) for the comparative molecular docking studies.
(TIF)

Table S1 Hot spot residues in CVN that target different ligands as determined by structural resolution approaches.
(DOCX)

## References

[1] UNAIDS website. Available: http://www.unaids.org/en/resources/documents/2012/name,76121,en.asp. Accessed 2013 Dec 18.

[2] Shattock RJ, Moore JP (2003) Inhibiting sexual transmission of HIV-1 infection. Nature Reviews Microbiology 1: 25–34. 15040177 10.1038/nrmicro729

[3] Shattock RJ, Warren M, McCormack S, Hankins CA (2011) AIDS. Turning the tide against HIV. Science 333: 42–43. 21719662 10.1126/science.1206399

[4] Balzarini J, Van Damme L (2007) Microbicide drug candidates to prevent HIV infection. Lancet 369: 787–797. 17336656 10.1016/S0140-6736(07)60202-5

[5] Bewley CA, Gustafson KR, Boyd MR, Covell DG, Bax A, et al (1998) Solution structure of cyanovirin-N, a potent HIV-inactivating protein. Nat Struct Biol 5: 571–578. 9665171 10.1038/828

[6] Xiong S, Fan J, Kitazato K (2010) The antiviral protein cyanovirin-N: the current state of its production and applications. Applied Microbiology and Biotechnology 86: 805–812. 20162270 10.1007/s00253-010-2470-1

[7] Zappe H, Snell ME, Bossard MJ (2008) PEGylation of cyanovirin-N, an entry inhibitor of HIV. Adv Drug Deliv Rev 60: 79–87. 17884238 10.1016/j.addr.2007.05.016

[8] Roberts MJ, Bentley MD, Harris JM (2002) Chemistry for peptide and protein PEGylation. Adv Drug Deliv Rev 54: 459–476. 12052709 10.1016/s0169-409x(02)00022-4

[9] Pasut G, Veronese FM (2012) State of the art in PEGylation: the great versatility achieved after forty years of research. J Control Release 161: 461–472. 22094104 10.1016/j.jconrel.2011.10.037

[10] Huston JS, Levinson D, Mudgett-Hunter M, Tai MS, Novotny J, et al (1988) Protein engineering of antibody binding sites: recovery of specific activity in an anti-digoxin single-chain Fv analogue produced in Escherichia coli. Proc Natl Acad Sci U S A 85: 5879–5883. 3045807 10.1073/pnas.85.16.5879 PMC281868

[11] Gao X, Chen W, Guo C, Qian C, Liu G, et al (2010) Soluble cytoplasmic expression, rapid purification, and characterization of cyanovirin-N as a His-SUMO fusion. Appl Microbiol Biotechnol 85: 1051–1060. 19547966 10.1007/s00253-009-2078-5 PMC7080120

[12] Tochikura TS, Nakashima H, Tanabe A, Yamamoto N (1988) Human immunodeficiency virus (HIV)-induced cell fusion: quantification and its application for the simple and rapid screening of anti-HIV substances in vitro. Virology 164: 542–546. 3369092 10.1016/0042-6822(88)90570-3

[13] Botos I, Mori T, Cartner LK, Boyd MR, Wlodawer A (2002) Domain-swapped structure of a mutant of cyanovirin-N. Biochem Biophys Res Commun 294: 184–190. 12054761 10.1016/S0006-291X(02)00455-2

[14] Vorontsov, II, Miyashita O (2009) Solution and crystal molecular dynamics simulation study of m4-cyanovirin-N mutants complexed with di-mannose. Biophys J 97: 2532–2540. 19883596 10.1016/j.bpj.2009.08.011 PMC2770623

[15] Bewley CA (2001) Rapid validation of the overall structure of an internal domain-swapped mutant of the anti-HIV protein cyanovirin-N using residual dipolar couplings. J Am Chem Soc 123: 1014–1015. 11456652 10.1021/ja005714o

[16] Sandstrom C, Hakkarainen B, Matei E, Glinchert A, Lahmann M, et al (2008) Atomic mapping of the sugar interactions in one-site and two-site mutants of cyanovirin-N by NMR spectroscopy. Biochemistry 47: 3625–3635. 18311923 10.1021/bi702200m

[17] Botos I, O’Keefe BR, Shenoy SR, Cartner LK, Ratner DM, et al (2002) Structures of the complexes of a potent anti-HIV protein cyanovirin-N and high mannose oligosaccharides. J Biol Chem 277: 34336–34342. 12110688 10.1074/jbc.M205909200

[18] Barrientos LG, Matei E, Lasala F, Delgado R, Gronenborn AM (2006) Dissecting carbohydrate-Cyanovirin-N binding by structure-guided mutagenesis: functional implications for viral entry inhibition. Protein Engineering Design and Selection 19: 525–535. 10.1093/protein/gzl040 17012344

[19] Corine Sandströ m, Berteau O (2005) Atomic Mapping of the Interactions between the Antiviral Agent Cyanovirin-N and Oligomannosides by Saturation-Transfer Difference NMR†. Biochemistry 43: 13926–13931. 10.1021/bi048676k 15518540

[20] Geyer H, Holschbach C, Hunsmann G, Schneider J (1988) Carbohydrates of human immunodeficiency virus. Structures of oligosaccharides linked to the envelope glycoprotein 120. J Biol Chem 263: 11760–11767. 2841333

[21] Montero M, van Houten NE, Wang X, Scott JK (2008) The membrane-proximal external region of the human immunodeficiency virus type 1 envelope: dominant site of antibody neutralization and target for vaccine design. Microbiol Mol Biol Rev 72: 54–84, table of contents. 10.1128/MMBR.00020-07 PMC2268283 18322034

[22] Shattock R, Solomon S (2004) Microbicides–aids to safer sex. Lancet 363: 1002–1003. 15051276 10.1016/S0140-6736(04)15876-5

[23] Moscoso CG, Sun Y, Poon S, Xing L, Kan E, et al (2011) Quaternary structures of HIV Env immunogen exhibit conformational vicissitudes and interface diminution elicited by ligand binding. Proc Natl Acad Sci U S A 108: 6091–6096. 21444771 10.1073/pnas.1016113108 PMC3076850

[24] Chen X, Zaro JL, Shen WC (2013) Fusion protein linkers: Property, design and functionality. Adv Drug Deliv Rev 65: 1357–1369. 23026637 10.1016/j.addr.2012.09.039 PMC3726540

[25] Liu Y, Carroll JR, Holt LA, McMahon J, Giomarelli B, et al (2009) Multivalent interactions with gp120 are required for the anti-HIV activity of Cyanovirin. Biopolymers 92: 194–200. 19235857 10.1002/bip.21173 PMC6961781

[26] Kelley BS, Chang LC, Bewley CA (2002) Engineering an obligate domain-swapped dimer of cyanovirin-N with enhanced anti-HIV activity. J Am Chem Soc 124: 3210–3211. 11916396 10.1021/ja025537m

[27] Contarino M, Bastian AR, Kalyana Sundaram RV, McFadden K, Duffy C, et al (2013) Chimeric Cyanovirin-MPER recombinantly engineered proteins cause cell-free virolysis of HIV-1. Antimicrob Agents Chemother 57: 4743–4750. 23856780 10.1128/AAC.00309-13 PMC3811417

[28] NIH. Available: http://chemdb.niaid.nih.gov/struct_search/misc/invitro.asp?aids=045707. Accessed 2012 May 12.

[29] Xiong S, Fan J, Kitazato K (2010) The antiviral protein cyanovirin-N: the current state of its production and applications. Appl Microbiol Biotechnol 86: 805–812. 20162270 10.1007/s00253-010-2470-1

[30] Boyd MR, Gustafson KR, McMahon JB, Shoemaker RH, O’Keefe BR, et al (1997) Discovery of cyanovirin-N, a novel human immunodeficiency virus-inactivating protein that binds viral surface envelope glycoprotein gp120: potential applications to microbicide development. Antimicrob Agents Chemother 41: 1521–1530. 9210678 10.1128/aac.41.7.1521 PMC163952

[31] Berman HM, Westbrook J, Feng Z, Gilliland G, Bhat TN, et al (2000) The Protein Data Bank. Nucleic Acids Res 28: 235–242. 10592235 10.1093/nar/28.1.235 PMC102472

[32] Nemoto Y, Ikeda J, Katoh K, Koshimoto H, Yoshihara Y, et al (1993) R2D5 antigen: a calcium-binding phosphoprotein predominantly expressed in olfactory receptor neurons. J Cell Biol 123: 963–976. 8227152 10.1083/jcb.123.4.963 PMC2200141

[33] Katoh M, Shikoshi K, Takada M, Umeda M, Tsukahara T, et al (1993) [Neutrophil functions during treatment with granulocyte colony-stimulating factor (G-CSF) in the elderly with non-Hodgkin’s lymphoma: including two patients accompanied with interstitial pneumonitis during the treatment with G-CSF]. Nihon Ronen Igakkai Zasshi 30: 953–957. 7507533 10.3143/geriatrics.30.953

[34] Nakashima H, Tanabe A, Tochikura TS, Yamamoto N (1988) Rapid screening method with a cell multisizer for inhibitors of human immunodeficiency virus-induced cell fusion in vitro. J Clin Microbiol 26: 1229–1232. 3384935 10.1128/jcm.26.6.1229-1232.1988 PMC266571

[35] Katoh H, Satomura S, Matsuura S (1993) Analytical method for sugar chain structures involving lectins and membrane ultrafiltration. J Biochem 113: 118–122. 7681055 10.1093/oxfordjournals.jbchem.a123994

[36] Buckheit RW Jr, Roberson JL, Lackman-Smith C, Wyatt JR, Vickers TA, et al (1994) Potent and specific inhibition of HIV envelope-mediated cell fusion and virus binding by G quartet-forming oligonucleotide (ISIS 5320). AIDS Res Hum Retroviruses 10: 1497–1506. 7888204 10.1089/aid.1994.10.1497

