# Supplementary figures and images for: Linker-Extended Native Cyanovirin-N Facilitates PEGylation and Potently Inhibits HIV-1 by Targeting the Glycan Ligand
Source: PLoS One. 2014 Jan 27;9(1):e86455. doi: 10.1371/journal.pone.0086455 (PMC3903522; doi:10.1371/journal.pone.0086455)

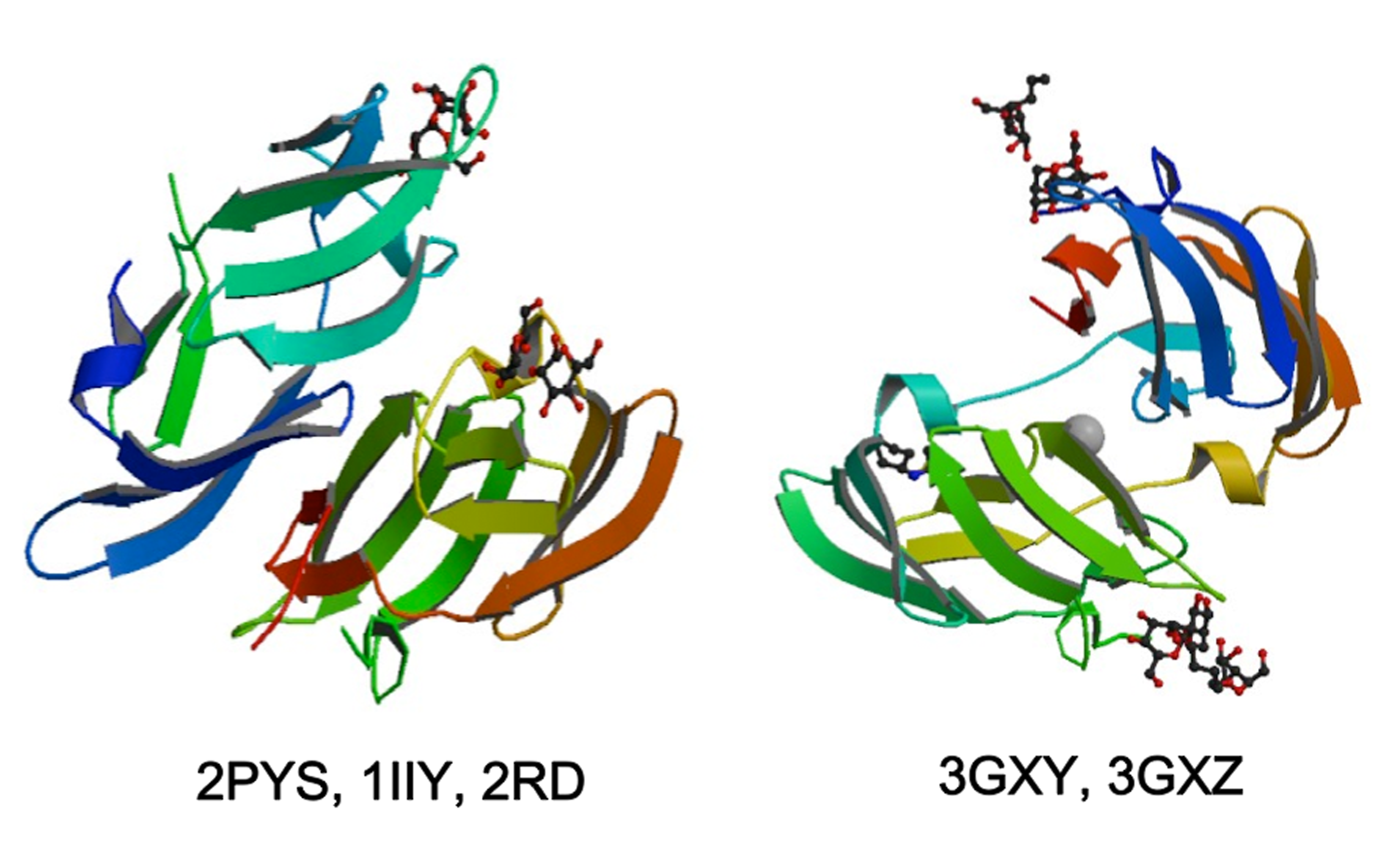

Supplement: Figure S1 — The three-dimensional (3D) structures of CVN utilized in this study. 2PYS, 1IIY and 2RDK are parallel domain-swapped dimers of CVN, and 3GXY and 3GXZ are reverse-parallel domain-swapped dimers of CVN. The structure coordinates of the protein-ligand complexes were retrieved from the Protein Data Bank (PDB) for the comparative molecular docking studies. (TIF) [file pone.0086455.s001.tif]
