# Supplementary material for: Linker-Extended Native Cyanovirin-N Facilitates PEGylation and Potently Inhibits HIV-1 by Targeting the Glycan Ligand
Source: PLoS One. 2014 Jan 27;9(1):e86455. doi: 10.1371/journal.pone.0086455 (PMC3903522; doi:10.1371/journal.pone.0086455)
Supplement: Table S1 — Hot spot residues in CVN that target different ligands as determined by structural resolution approaches. (DOCX) [file pone.0086455.s002.docx]

**Table S1 Hot spot residues in CVN that target different ligands as determined by structural resolution approaches**

| Structure | Analysis method | Ligands | Hot spot residues* |
| --- | --- | --- | --- |
| 3GXY 3GXZ | Docking | No. 16-17, 19-22 | Leu-1, Gly-2, Lys-3, Gln-6  Thr-7, Tyr-9, Glu-23, Thr-25  Gly-27, Asn-93, Asp-95, Glu-101 |
|  | Crystallography | OPM-MAN-MAN NAG-NAG-BMA-MAN-MAN MAN-MAN, MAN-MAN | Gly-2, Lys-3, Thr-7, Glu-23  Asp-95, Asn-93 |
| 2PYS 1IIY 2RDK | Docking | No. 16-17, 19-22 | Glu-41, Asn-42, Ser-52, Asn-53  Glu-56, Thr-57, Lys-74, Arg-76 |
|  | Crystallography | MAN-MAN, MAN-MAN MAN-MAN, MAN-MAN | Asn-42, Asp-44, Ser-52, Asn-53  Thr-57, Lys-74, Gln-78 |

No. 16-17 and 19-22, high mannose oligosaccharides listed in Figure 3;

OPM-MAN-MAN, NAG-NAG-BMA-MAN-MAN and MAN-MAN are ligands utilized for CVN crystallography.

*The residues identified by both docking and crystallography are underlined.
